# Study on the mechanism of acupuncture to improve mild cognitive impairment in hypertension by regulating intestinal microbiome

**DOI:** 10.3389/fnins.2024.1495384

**Published:** 2024-12-23

**Authors:** Xinlei Dong, Xiaomin Hao, Jian Wen, Qinfeng Yan, Kaixuan Ma, Qingguo Liu, Juan Li, Lili Zhang

**Affiliations:** ^1^First Teaching Hospital of Tianjin University of Traditional Chinese Medicine, Tianjin, China; ^2^National Clinical Research Center for Chinese Medicine Acupuncture and Moxibustion, Tianjin, China; ^3^Dongzhimen Hospital, Beijing University of Chinese Medicine, Beijing, China; ^4^School of Acupuncture, Moxibustion and Tuina, Beijing University of Chinese Medicine, Beijing, China; ^5^Chengdu University of Traditional Chinese Medicine, College of Health Preservation and Rehabilitation, Chengdu, China; ^6^Affiliated Rehabilitation Hospital of Chengdu University of Traditional Chinese Medicine, Chengdu, China

**Keywords:** acupuncture, gut microbiota-brain-gut axis, hypertension, mild cognitive impairment, intestinal flora

## Abstract

High blood pressure is a significant risk factor for cardiovascular diseases and is linked to an increased risk of mild cognitive impairment (MCI). The lack of effective treatments for these conditions highlights the urgent need for novel therapeutic approaches. Recent research suggests that the gut microbiota-brain-gut axis plays a crucial role in the pathogenesis of hypertension and MCI by regulating the nervous, endocrine, and immune systems. Acupuncture, an established therapeutic modality, has shown promise in influencing the course of hypertension and MCI by modulating the gut microbiota. This review aims to summarize the mechanistic relationships between the gut microbiome, hypertension, and MCI, and to explore the potential of acupuncture as a treatment strategy for managing Mild cognitive impairment in Hypertension concurrently.

## 1 Introduction

Hypertension is a major risk factor for cardiovascular disease and significantly contributes to the global burden of morbidity and mortality (Pazoki et al., 2018). According to global hypertension epidemiology data, 31.1% of adults worldwide (approximately 1.39 billion people) had high blood pressure in 2010 (Mills et al., 2020). Between 2014 and 2017, the prevalence of hypertension in China was reported to be as high as 44.7% (Lu et al., 2017). MCI is defined as a condition that represents a transitional state between normal cognitive function and dementia (Chen Y. X. et al., 2021). It is characterized by a mild decline in cognitive functions such as memory and thinking, without significantly impacting the ability to perform daily activities (Qin et al., 2021). According to statistics, currently, 15.5% of Chinese adults have MCI, and the annual conversion rate of MCI to dementia is approximately 10–20% (Etgen et al., 2011). MCI and dementia share several common risk factors, including hypertension, hyperlipidemia, diabetes, and cardiovascular and cerebrovascular diseases (Jia et al., 2020). An increasing number of studies confirm a strong association between high blood pressure and mild cognitive impairment, vascular dementia, and Alzheimer’s disease (Nagai et al., 2010; Zamora and Williamson, 2020; Shajahan et al., 2024). A 2021 meta-analysis found that approximately 30% of individuals with high blood pressure had MCI (Qin et al., 2021), and high blood pressure increased the risk of MCI by 1.14 times.

Currently, no drugs are approved by the U.S. Food and Drug Administration (FDA) for the treatment of MCI (Jia et al., 2020). Therefore, it is crucial to thoroughly understand the complex pathological mechanisms linking hypertension and MCI and to identify effective treatment strategies. The intestinal microbiota is primarily composed of Firmicutes, Bacteroidetes, Actinobacteria, Proteobacteria, Clostridia, and Verrucomicrobia, with Bacteroidetes and Firmicutes being the predominant members (Hou et al., 2022). These microorganisms play a crucial role in maintaining homeostasis and regulating the functions of various bodily systems (Cryan et al., 2020). Studies have shown that alterations in the gut microbiota are strongly associated with various symptoms and diseases, including pain (Guo et al., 2019), autism (Li Q. et al., 2017), neurodegenerative disease (Quigley, 2017), and cerebrovascular diseases (Honarpisheh et al., 2022) among others.

**FIGURE 1:**
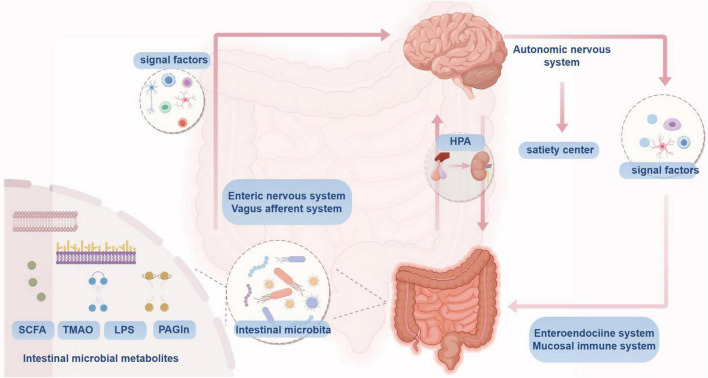
Microbial-gut-brain axis conceptual mechanism.

Intestinal microorganisms establish bidirectional communication with the central nervous system (CNS)through neural, endocrine, immune, and other pathways, collectively known as the microbiota-gut-brain axis (Cryan et al., 2020). On one hand, the CNS can influence the composition and ecological environment of intestinal microorganisms through the central and autonomic nervous systems, the hypothalamic-pituitary-adrenal (HPA) axis, and the release of related signaling factors. On the other hand, gut microbes can directly regulate metabolic and immune signaling via neural pathways, including the enteric nervous system (ENS) and the vagus afferent nerve (VAN), as well as endocrine pathways represented by the HPA axis, thereby affecting CNS function (Collins et al., 2012; Wang and Kasper, 2014), as illustrated in Figure 1. There are several common pathological mechanisms between hypertension and MCI, such as neuroendocrine dysfunction (O’Caoimh et al., 2014), inflammation and immune responses (Cho, 2023; Gan et al., 2024), including the microbial-gut-brain axis.

Acupuncture, a specialized treatment modality in traditional Chinese medicine, has been proven to improve hypertension through multiple pathways and targets. Studies have shown that acupuncture influences neuro (Fan et al., 2020)-endocrine (Lee and Kim, 1994) function, immune inflammation (Chang Lee et al., 2016), redox balance (Tanaka and Laurindo, 2018; Wang et al., 2018), endothelial function (Leung et al., 2016), and other aspects through multiple pathways (Dong et al., 2024) and targets (Li et al., 2021), thereby helping to lower blood pressure. Acupuncture also plays a role in enhancing cognitive function (Tian et al., 2013).

This article will explore the association between gut microbiota, hypertension, and cognitive impairment, as well as the effects of acupuncture on regulating gut microbiota in the treatment of hypertension and the enhancement of cognitive function. Current studies have shown that gut microbes are closely associated with hypertension and cognitive impairment (Meyer et al., 2022; Qu et al., 2022; Zhou et al., 2023). Acupuncture is believed to regulate gut microbes, thereby exerting positive effects on the treatment of hypertension and enhancing cognitive function. However, there is a lack of systematic reviews and in-depth discussions in this field. Therefore, this article will summarize the mechanisms linking the microbiota-gut-brain axis, hypertension, and mild cognitive impairment, as well as the research progress on how acupuncture modulates this association. This study aims to explore the regulatory mechanisms of acupuncture and the intestinal microbiome on hypertension complicated with mild cognitive impairment, and to provide new insights into non-drug therapies for this condition, addressing the current lack of effective treatment strategies for mild cognitive impairment.

## 2 Hypertension and microbiome - gut - brain axis

**FIGURE 2:**
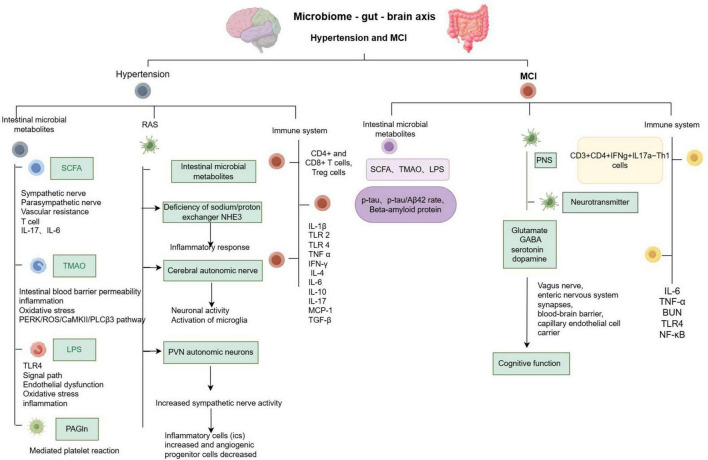
Summary of microbiome - gut - brain axis - related pathological mechanisms in hypertension and mild cognitive impairment.

Dysregulation of the gut microbiota is strongly associated with hypertension and is characterized by several changes, including an altered Firmicutes to Bacteroidetes ratio (F/B ratio), a reduction in short-chain fatty acid (SCFA)-producing bacteria, an increase in lactic acid bacteria, a decrease in Bacteroidetes, and an increase in Proteobacteria and Cyanobacteria (Verhaar et al., 2020). Additionally, the richness and diversity of intestinal microorganisms are reduced (Yang et al., 2015). Current studies have indicated a bidirectional relationship between gut microbiota and blood pressure regulation: hypertension can alter the structure of the gut microbiota (Zubcevic et al., 2019; Xia et al., 2021), and gut microbiota can, in turn, influence blood pressure regulation (Li J. et al., 2017; Yang and Zubcevic, 2017; Marques et al., 2018; de Almeida Silva et al., 2020; Yan et al., 2020; Gao et al., 2024), as shown in Figure 2.

### 2.1 Hypertension and intestinal microbial metabolites

Key metabolites produced by the gut microbiota include short-chain fatty acids (SCFAs), trimethylamine (TMA), lipopolysaccharide (LPS), and phenylacetylglutamine (PAGln) (Chen et al., 2023). These metabolites influence host blood pressure through various pathways, including the sympathetic nervous system, immune system, inflammatory response, renin- RAS, and gut-blood barrier. Collectively, these pathways play a role in the regulation of host blood pressure levels.

#### 2.1.1 Short-chain fatty acids (SCFA)

Short-chain fatty acids, primarily acetate, propionate, and butyrate, are produced by the fermentation of fibrous substances by gut bacteria. These SCFAs regulate host physiological functions by acting as ligands for host G protein-coupled receptors (GPCRs), including GPR41, GPR43, OLFR78, and GPR109A (Hu et al., 2022).

Natarajan et al. (2016) demonstrated that the SCFA receptor GPR41, primarily located in endothelial cells, plays a key role in mediating SCFA-induced vasodilation, enhancing sympathetic drive to regulate baseline blood pressure, and reducing salt-sensitive blood pressure. Dardi et al. (2022) used gas chromatography, PCR quantitative analysis, and pressure myogram analysis to find that, compared with normotensive rats, the mRNA expression of SCFA receptors in the mesenteric resistance arteries (MRA) of spontaneously hypertensive rats (SHR) is reduced. This reduction indicates decreased utilization of butyrate, an increased peripheral vascular resistance index (W/L), and vascular remodeling, which collectively contribute to elevated peripheral vascular resistance and increased blood pressure (Dardi et al., 2022).

SCFAs have anti-inflammatory effects and play a role in immune regulation in hypertension. In hypertension, natural killer cells and helper T cells (Th17) produce the pro-inflammatory cytokine IL-17. SCFAs regulate immunity by inhibiting histone deacetylase (HDAC) in T cells, promoting the acetylation of kinase p70S6 and phosphorylated rS6, which subsequently activates the mTOR pathway essential for Th17 cell proliferation, thereby potentially exacerbating hypertension (Yang et al., 2022). In addition, treatment with SCFAs has been shown to prevent vascular dysfunction and elevated blood pressure (BP) in mice with systemic lupus erythematosus (SLE) induced by imiquimod (IMQ) activation of TLR7. This protective effect is primarily observed through the reduction of left ventricular hypertrophy, enhancement of endothelium-dependent aortic relaxation, reduction of NADPH oxidase activity, and decreased Th17 cell infiltration, thereby contributing to vascular protection. SCFAs can also counteract the phenotype transfer of hypertension caused by intestinal microbial inoculation. Specifically, butyrate effectively reduces the expression of the IL-6 receptor, thereby inhibiting Th17 cell polarization, while acetate inhibits the accumulation of CX3CR1 + cells in mesenteric lymph nodes (MLNs) and reduces IL-6-driven Th17 polarization, playing a crucial role in intestinal immune regulation (Moleón et al., 2023). In addition, animal studies have confirmed that SCFAs can reduce blood pressure through activation of the parasympathetic nervous system (Onyszkiewicz et al., 2019; Yang et al., 2019; Verhaar et al., 2020; Tilves et al., 2022).

#### 2.1.2 Trimethylamine (TMA)

Diets containing choline compounds, such as choline, L-carnitine, betaine, and phosphatidylcholine, can be converted into TMA in the colon by gut microbiota. After absorption in the intestine, TMA is transported to the liver, where it is oxidized by flavin-containing monooxygenase 3 (FMO3) to form trimethylamine N-oxide (TMAO) (He S. et al., 2021). In hypertensive patients, gene expression and FMO protein levels are elevated in the liver, leading to an accelerated rate of TMA oxidation to TMAO (Gawryś-Kopczyńska et al., 2024). A meta-analysis on the association between TMAO and health has demonstrated that TMAO can serve as a novel biomarker, with its concentrations positively correlated with the risk of cardiovascular disease (Li et al., 2022).

Jaworska et al. (2017) found that, compared with normotensive rats, intestinal TMA permeability was increased in SHR, which affected intestinal morphology and hemodynamics. These results suggest that hypertension may enhance the permeability of the gut-blood barrier, thereby impacting the permeability of the intestinal microbial circulatory system (Jaworska et al., 2017). Huc et al. (2018) found that low-dose TMAO treatment improved hemodynamic, biochemical, and histopathological manifestations of cardiomyocyte hypertrophy and cardiac fibrosis in SHRs. In addition, Liu et al. (2022) demonstrated that inhibiting TMAO production reduces neuroinflammation and oxidative stress in the paraventricular nucleus (PVN) of the hypothalamus, alleviates sympathetic excitation, and prevents hypertension in rats fed a high-salt diet. Elevated plasma TMAO levels are associated with increased levels of pro-inflammatory cytokines, including IL-1β, IL-18, and TNF-α, and a decreased expression of the anti-inflammatory cytokine IL-10. In addition, evidence suggests that TMAO can enhance angiotensin II (Ang II)-induced vasoconstriction by activating the PERK/ROS/CaMKII/PLCβ3 pathway, thereby influencing the release of Ca^2+^ (Jiang et al., 2021); TMAO regulates macrophage receptors, promotes arteriosclerosis and vasoconstriction, interferes with cholesterol transport, promotes the production of pro-inflammatory and anti-inflammatory factors, and induces renal fibrosis, all of which contribute to alterations in host blood pressure levels (Mutengo et al., 2023).

#### 2.1.3 Lipolyaccharide (LPS)

Lipolyaccharide, a component of Gram-negative bacteria such as *E. coli*, is closely linked to the development of ecological disorders associated with high blood pressure. Grylls et al. (2021) demonstrated that LPS activates a signaling pathway through Toll-like receptor 4 (TLR4) in endothelial cells. This activation triggers several pathways, including the nicotinamide adenine dinucleotide phosphate (NADPH) oxidase/reactive oxygen species (ROS)/endothelial nitric oxide synthase (eNOS) pathways, mitogen-activated protein kinase (MAPK) pathways, and nuclear factor κB (NF-κB) pathways. These pathways contribute to endothelial dysfunction, vascular inflammation, and ultimately, hypertension. Probiotic supplementation has been shown to improve blood vessel relaxation, reduce vascular inflammation, and lower blood pressure (Grylls et al., 2021). In rats on a high-salt diet, there is an increase in peroxisome activity and LPS biosynthesis in the gut microbiome. This increase may exacerbate oxidative stress, promote apoptosis, and lead to liver and kidney dysfunction and metabolic disturbances, thereby contributing to the development of hypertension (Ou-Yang et al., 2022).

Additionally, the immune system is closely linked to Toll-like receptor (TLR) signaling, which plays a significant role in the development of hypertension. Specifically, in experimental models of hypertension, TLR4 has been shown to promote vascular dysfunction and contribute to the pathogenesis of hypertension (Goulopoulou et al., 2016). Our findings indicate that TLR4 mRNA levels are elevated in the aorta of SLE mice. Furthermore, in the vasculature, TLR4 is activated by bacterial products such as LPS, leading to vascular oxidative stress, inflammation, and endothelial dysfunction, which in turn affects blood pressure in these models (Toral et al., 2019).

#### 2.1.4 Phenylacetylglutamine (PAGln)

Studies have shown that PAGln is a metabolite dependent on the intestinal microbiome. It is primarily derived from dietary protein phenylalanine (Phe), which is converted to phenylacetic acid (PAA) by gut bacteria. PAA is then absorbed into the portal vein circulation and subsequently combined with glutamine by enzymes in the liver and kidneys to form PAGln (Zhu et al., 2023). Hypertension is a hemodynamic disorder characterized by abnormal vascular pressure and blood flow, which can lead to various thrombotic complications. One of the most notable features of hypertension is abnormal platelet activation (Zhao et al., 2021). Current research indicates that *in vitro* studies using whole blood, platelet-rich plasma (PRP), and isolated platelets have demonstrated that PAGln enhances the platelet response to multiple agonists and stimulates intracellular calcium release, thereby increasing platelet function. PAGln mediates platelet response and thrombosis through G protein-coupled receptors, such as α2A, α2B, and β2-adrenergic receptors. Therefore, it can be concluded that PAGln is correlated with hypertension (Nemet et al., 2020).

### 2.2 Hypertension, gut microbes, and the renin-angiotensin system

Research indicates that the RAS comprises multiple angiotensin peptides that mediate various biological functions through distinct receptors. Key components of the RAS include angiotensinogen, renin, angiotensin (Ang) I, AngII, angiotensin-converting enzyme (ACE), and ACE2. Among these, AngII is known to influence the onset and progression of hypertension through the AngII type 1 receptor (AT1R). ACE helps mitigate the harmful effects of AngII and plays a role in regulating intestinal microbiome disorders as well as local and systemic immune responses. Studies have demonstrated that intestinal microbial metabolites, acting as bioregulators, can mediate the pathological processes associated with AngII-induced hypertension. Additionally, AngII can directly or indirectly disrupt the intestinal microbiome in hypertensive patients (Li et al., 2024). In the mouse gut, AngII induces the loss of the sodium/proton exchanger NHE3, leading to increased intraluminal pH, inflammatory responses, and microbial dysbiosis (Larmonier et al., 2013; Li X. C. et al., 2019). Analysis of plasma and fecal samples using liquid chromatography-tandem mass spectrometry revealed that conventional mice implanted with AngII exhibited 25 significantly upregulated metabolites and 71 significantly downregulated metabolites compared to germ-free mice, with notable downregulation in AngII-treated mice. These findings suggest that AngII influences intestinal ecology by modulating metabolites through the intestinal microbiome (Cheema and Pluznick, 2019).

Additionally, in SHR rats treated with an ACE inhibitor peptide for 4 weeks, the intestinal microbiota resembled that of normal control rats. This improvement was characterized by a restoration of Firmicutes abundance, a reduction in Trichospirillaceae and Ruminococcaceae, and an increase in SCFA-producing flora, such as Lactobacillus. These changes restored the diversity of the intestinal microbiota and helped prevent hypertension-related microbial disorders (Xie et al., 2022). The autonomic nervous system is intricately connected with the intestinal system, and AngII triggers increased neuronal activity and activation of resident microglia in autonomic brain regions. This, in turn, enhances sympathetic nervous system (SNS) activity, which affects epigenetic changes in the gut and influences gut microbiota. Furthermore, AngII activates PVN autonomic neurons, increasing sympathetic nerve activity and impacting the bone marrow. This results in an increase in inflammatory cells (IC) and a decrease in angiogenic progenitor cells, ultimately leading to elevated blood pressure. ICs may migrate to the brain, transform into microglia/macrophages, and propagate neuroinflammation, causing pathological changes in the gut (Liu W. et al., 2023). Inhibiting microglial activation in the PVN can reduce sympathetic nerve activity and ameliorate hypertensive intestinal histopathology (Sharma et al., 2019). In summary, there is a bidirectional regulatory effect between gut microbes and the RAS.

### 2.3 Hypertension, gut microbes, and the immune system

The immune system can be categorized into innate and adaptive immunity. The gut microbiota interacts with both components of the immune system to serve as the first line of defense against microbial invasion, while also preventing bacterial translocation and infection (Campbell et al., 2023). Disruption of gut flora can trigger inflammatory responses in damaged organs by activating immune cells and releasing pro-inflammatory cytokines, thereby exacerbating cardiovascular conditions such as hypertension. Furthermore, metabolites released due to an imbalance in the gut microbiota can promote inflammation by altering the phenotype and function of immune cells and activating inflammation-related pathways (Nesci et al., 2023; Wang et al., 2023).

Evidence of immune system impairment has been observed in germ-free mice, including reduced T cell numbers, impaired function of CD4 + and CD8 + T cells, Treg cells, and compromised antibody production. Recolonization of gut microbes has been shown to restore these immune functions (Santisteban et al., 2017). In rats with hypertension, not only were changes in gut flora observed, but there was also an increase in gut inflammation. This was evidenced by elevated mRNA levels of interleukin-1β (IL-1β), high mobility group box 1 (HMGB1), tumor necrosis factor α (TNF-α), Toll-like receptors 2 (TLR2) and 4 (TLR4), and advanced glycation end-product receptor (RAGE) in the small intestine of SHRs. This increase is attributed to the activation of immune cells and an increase in bone marrow ICs in the gut (Santisteban et al., 2017).

At the same time, ANGII-induced vascular inflammation was reduced in germ-free mice. This reduction was evidenced by significant decreases in the signature cytokines IFN-γ, IL-4, and IL-10 in the splenic supernatant, which were restored in mice colonized with gut microbes. Additionally, enhanced vasoconstriction and mild endothelial dysfunction were observed in the mice transplanted with gut microbiota (Karbach et al., 2016). Hypertensive mice on a high-salt diet exhibited reduced abundance of Lactobacillus and increased levels of interleukin-17A CD4 + helper T cells (Th17) compared to mice on a normal salt diet. Supplementation with Lactobacillus led to a decrease in Th17 cells, which helped improve salt-sensitive hypertension (Wilck et al., 2017). Studies have shown that the expression or secretion of numerous immune factors, such as IL-17, MCP-1, IL-6, TGF-β, and IL-10, is regulated by the microbiome or its metabolites, potentially impacting hypertension (Yang et al., 2022). Correlation analysis revealed that increased levels of IL-1β, IL-6, IL-8, and TNF-α in hypertensive rats were significantly correlated with the abundance of intestinal microbiota, particularly with subspecies of Bifidobacterium (Zheng et al., 2023). In conclusion, the regulation of the inflammatory response represents a critical link between gut microbiota and hypertension.

## 3 MCI and microbiome - gut - brain axis

MCI is considered a precursor to Alzheimer’s disease (AD). 16S rRNA gene sequencing has revealed that the abundance of gut microbiota is closely related to cognitive function (Pei et al., 2023). Comparisons of microbial communities in the stool and blood of AD patients, MCI patients, and healthy controls showed no significant differences between AD and MCI. In the stools of AD and MCI patients, there was an increase in E. coli and Lactobacillus, while Bacteroides decreased. Quantitative 16S microarray technology demonstrated that the changes in Bacteroides in MCI were consistent with those observed in AD, while changes in Staphylococcus were associated with neurodegeneration (Pan et al., 2021). Furthermore, the amyloid pathogenesis of AD may be initiated by the transfer of intestinal microbiota during MCI, suggesting that MCI exhibits similar changes in gut microbiota as seen in AD (Li B. et al., 2019). Current research indicates that the intestinal microbiota can regulate cognitive function through its metabolites, the nervous system, the immune system, and other pathways, as illustrated in Figure 2. The correlation between intestinal microbiota and AD is detailed in Table 1.

**TABLE 1:** The association between intestinal microbes and AD.

Serial number	Test object	Detection method	Relevant mechanism	Link
1	AD model mice constructed by injecting Aβ	16S rDNA Sequencing, Immunofluorescence, Western Blot	**Dysregulation of Intestinal Flora and Cholinergic Anti-Inflammatory Pathways:** Dysregulation of the intestinal microbiota inhibits cholinergic anti-inflammatory pathways, which can contribute to inflammation and cognitive impairment.	Qian et al., 2022
2	Patients with AD or cognitive impairment	Amyloid Protein PET, Microbial DNA qPCR	**Association Between Gut Flora and Cognitive Impairment:** The abundance of intestinal flora is linked to peripheral inflammatory states and brain amyloidosis in cognitive impairment, indicating a role in both systemic and CNSinflammation.	Cattaneo et al., 2017
3	AD patients, 11-week-old adult male Sprague-Dawley rat	16S Microbiota Analysis, Swiss-Roll Technology, MS-OMIC Metabolomics Analysis	**Gut Flora and Hippocampal Neurogenesis:** Gut flora influences hippocampal neurogenesis, thereby regulating systemic circulation and the centralization of CNScells.	Grabrucker et al., 2023
4	App knock-in (KI)mouse (AppNL-G-F)	Immunohistochemistry, Aβ ELISA, Western Blotting, Immunofluorescence Staining	**Effect of Bacillus Brevis MCC1274 Supplementation:** Supplementation with Bacillus brevis MCC1274 reduced Aβ plaque load in the hippocampus, diminished microglial activation, decreased mRNA expression levels of IL-1β and IL-6, and increased mRNA levels of TGF-β1 and synaptic proteins.	Abdelhamid et al., 2022
5	Cognitively normal patients without amyloid-beta (Aβ) accumulation (Aβ-NC) patients with Aβ positive mild cognitive impairment (Aβ + MCI)	18F-Florbetaben PET, Sequencing of Fecal Bacteria 16S rRNA Gene	**Butyrate-Producing Bacteria and Aβ Deposition:** A decrease in butyrate-producing bacteria is associated with increased Aβ deposition in the brain, which triggers a host immune response and enhances intestinal mucosal barrier function.	Kim et al., 2024
6	Subjects with normal Aβ-negative cognition (CN-), Subjects with normal Aβ-positive cognition (CN+) Patients with MCI and AD	Illumina MiSeq Sequencing	**Correlation Between Gut Flora and Aβ Load:** A higher relative abundance of Bacteroidetes and a decrease in Firmicutes and Proteobacteria are positively correlated with Aβ load and negatively correlated with plasma Aβ levels.	Sheng et al., 2022
7	5XFAD (Tg) mice and coresident WT mice (Corresponding WT mice produced by mating Tg mice and C57 mice)	DNA Extraction, PCR Amplification and Sequencing, Immunohistochemistry, Amino Acid Detection	**Phe and Isoleucine Accumulation:** Changes in intestinal flora lead to peripheral accumulation of phenylalanine and isoleucine, which stimulate the differentiation and proliferation of pro-inflammatory Th1 cells and activate M1 microglia, contributing to AD-related neuroinflammation.	Wang et al., 2019
8	Healthy middle-aged and elderly people	16S rRNA Amplicon, Whole-Genome Shotgun (WGS) Sequencing	**Gut Flora and Inflammatory Response:** A decrease in the relative abundance of Bacteroides, along with an increase in Prevotella, promotes inflammation. P. ruminicola enhances the production of SCFAs, regulates plasma metabolites, and affects neurotransmitter production, such as glutamate. High abundance of B. staiotaomicron can be an early indicator of AD.	Aljumaah et al., 2022
9	3 × Tg mouse	Microbial 16S rRNA sequencing	**Butyrate-Producing Bacteria and Neuroinflammation:** The absence of anti-inflammatory butyrate-producing bacteria in the AD gut microbiota, combined with elevated inflammatory PGE2 metabolites in the brain, increases microglial activation. This leads to chronic neuroinflammation and activates the C/EBP-β/AEP pathway, enhancing APP and tau expression levels and AEPδ-secretase activity, contributing to AD pathogenesis.	Chen C. et al., 2022
10	AD mouse	16S Ribosomal RNA (rRNA) Gene Amplification Sequence	**Phe and Isoleucine’s Role in Neuroinflammation:** Changes in gut flora result in peripheral accumulation of Phe and isoleucine, which stimulate the differentiation and proliferation of pro-inflammatory Th1 cells. This, in turn, activates M1 microglia, leading to AD-related neuroinflammation.	Wang et al., 2019
11	wild-type (WT) mice and AppNL-G-F AD mice	HEK-Blue mTLR4, RNA Extraction and RT-qPCR Analysis	**Helicobacter pylori-Derived Outer Membrane Vesicles and Cognitive Decline:** Outer membrane vesicles (OMVs) derived from Helicobacter pylori (H. pylori) can cross biological barriers and enter the brain. They interact with astrocytes, microglia, and neurons via the complement component 3 (C3) - C3a receptor (C3aR) signaling pathway, inducing glial cell activation and neuronal dysfunction. This interaction contributes to the pathological progression of amyloid beta and cognitive decline.	Xie et al., 2023
12	AD-like pathology with amyloid and neurofibrillary tangles (ADLPAPT) transgenic AD mice and healthy wild-type (WT) mice	Microbial 16S rRNA sequencing	**Early Life Changes in Gut Microbiota and Cognitive Impairment:** Alterations in gut microbiota composition early in life in ADLPAPT mice lead to chronic intestinal inflammation and loss of epithelial integrity, resulting in systemic inflammation. This is closely linked to Aβ deposition, tau pathology, reactive gliosis, monocyte recruitment, intestinal macrophage dysfunction, and cognitive impairment. Colonization with normal microbiota significantly mitigates these pathogenic characteristics.	Kim et al., 2020
13	Wild type and mutant amyloid precursor protein/prosenolin 1 (APP/PS1) mice	Microbial 16S rRNA sequencing	**Fecal Microbiota Transplantation (FMT) and Neuroinflammation:** Fecal microbiota transplantation (FMT) in elderly APP/PS1 mice increased intestinal BACE1 and Aβ42 levels. Intestinal Aβ42 was transmitted to the brain through the bloodstream, activating microglia and enhancing neuroinflammation. This process contributes to the development of Alzheimer’s disease (AD).	Jin J. et al., 2023
14	Mice receiving fecal microbiota transplantation (FMT) from a healthy donor, Thy1C/EBP-β transgenic mice pretreated with an antibiotic mixture	Microbial 16S rRNA sequencing	**Metabolites from Bacteroides Fragile and Neuroinflammation:** Stool samples from AD patients contain Bacteroides fragilis and its metabolites, including 12-hydroxy-heptatridecanoic acid (12-HHTrE) and prostaglandin E2 (PGE2). These metabolites upregulate the C/EBP-β/Asparagine endopeptidase (AEP) pathway, which is associated with microglial activation. This leads to the aggregation of Aβ and tau proteins and widespread neuroinflammation, accelerating cognitive impairment.	Xia et al., 2023

### 3.1 MCI and intestinal microbial metabolites

Measurements of TMAO levels in the cerebrospinal fluid (CSF) of patients with Alzheimer’s disease (AD), MCI, and cognitively unimpaired controls revealed that both MCI and AD dementia patients had higher CSF TMAO levels. Additionally, CSF TMAO levels were found to be positively correlated with CSF phosphorylated tau (p-tau), a biomarker of neuronal degeneration, and the p-tau/Aβ42 ratio (Vogt et al., 2018). Free water (FW) imaging, which estimates extracellular water content, has been used to study neuroinflammation in neurological diseases, including MCI and AD. Yamashiro et al. (2024) discovered that an increase in FW in gray matter regions (such as the frontal, temporal, limbic, and parietal marginal regions) associated with cognitive impairment in the AD/MCI group was correlated with a reduced abundance of butyrate-producing bacteria. The abundance of butyrate showed a significant decreasing trend across the groups, with the normal group having the highest levels, followed by the MCI group, and the AD group having the lowest levels. This indicates a correlation between neuroinflammation and decreased levels of the short-chain fatty acid butyrate (Yamashiro et al., 2024). Observations of aged rats with MCI and AD revealed significantly increased levels of serum tau protein, β-amyloid protein, and LPS with age. This was accompanied by an increased abundance of E. coli in the intestinal flora, which promoted amyloid protein aggregation and neurofibrillary tangle formation, exacerbating cognitive dysfunction (Sánchez-Tapia et al., 2023). In summary, intestinal microbial metabolites may influence cognitive function by regulating the distribution of cognitive-related proteins and modulating inflammatory factors.

### 3.2 MCI, gut microbes, and the nervous system

Previous studies have demonstrated that gut microbes play a role in maintaining cognitive function by linking the gut-brain axis with the parasympathetic nervous system (PNS). Park and Wu (2022) found that scopolamine injection, which inhibits the PNS and induces memory deficits, primarily affects the intestinal microbiota by decreasing Bacteroides and increasing Clostridium, Lactobacillus, and Escherichia coli. This PNS inhibition leads to dysregulation of the intestinal microbiota, enhanced inflammation, increased sympathetic nerve activity, exacerbation of cognitive impairment, and progression from MCI to Alzheimer’s disease (AD) (Park and Wu, 2022). Studies have also shown that intestinal microbiota metabolites can act as signaling molecules, prompting intestinal endocrine cells to synthesize or release neurotransmitters such as glutamate, GABA, serotonin, and dopamine. These metabolites influence the brain and peripheral systems through various pathways, including the vagus nerve, ENS synapses, the blood-brain barrier, capillary endothelial cell carriers, and local serotonin signal regulation, thereby affecting cognitive function (Chen Y. et al., 2021).

### 3.3 MCI, gut microbes, and the immune system

Previous studies have identified that in MCI rats induced by AlCl3, the relative abundance of the Pectinophilus group is low and negatively correlated with inflammatory factors, while the relative abundance of Blautia is negatively correlated with the expression of inflammatory proteins such as IL-6, TNF-α, and BUN (Chen S. Y. et al., 2022). The SAMP8 (Accelerated Aging Mouse Prone Position 8) model is commonly used to study MCI, and Probiotic-4 is a formulation consisting of *Bifidobacterium lactis*, *Lactobacillus casei*, *Bifidobacterium bifidum*, and *Lactobacillus acidophilus*. Research has shown that oral administration of Probiotic-4 can alleviate age-related disruptions to the intestinal barrier and blood-brain barrier, reduce mRNA and protein levels of interleukin-6 and tumor necrosis factor α, decrease plasma and brain LPS concentrations, and lower the expression of Toll-like receptor 4 (TLR4). Additionally, Probiotic-4 reduces the nuclear translocation of nuclear factor-κB (NF-κB) in the brain. By eliminating retinoic acid-inducible gene I (RIG-I) polyubiquitination in the brain and inhibiting the TLR4 and RIG-I-mediated NF-κB signaling pathways and inflammatory responses, Probiotic-4 improves the gut-brain axis and ultimately plays a neuroprotective role associated with cognitive impairment (Yang et al., 2020).

The mechanism by which cognitive impairment occurs due to ketogenic diet and hypoxia involves changes in the gut microbiota, specifically Bilophila. This microbial alteration stimulates the expansion of Th1 cells, which in turn affects hippocampal function and leads to cognitive impairment. Additionally, evidence indicates that colonization by B. wadsworthia promotes the expansion of IFN-γ-producing T-helper type 1 (Th1) cells. This is evidenced by increased levels of CD3 + CD4 + IFN-γ + IL-17a-Th1 cells in the lamina propria of the colon. Such changes are closely linked to physiological homeostasis and cognitive function in the hippocampus (Olson et al., 2021).

## 4 Acupuncture improves hypertension with MCI by regulating microbial-gut-brain axis

### 4.1 Acupuncture improves cognitive function by lowering blood pressure

Some studies have indicated that combinations of acupuncture points (LR3 + KI3) or individual points (LR3 or KI3) can help lower blood pressure. Interestingly, the effect of combining acupuncture points is not merely additive but synergistic. This synergistic effect influences multiple brain regions, including the somatosensory cortex, premotor cortex, and others, which are closely associated with cognitive functions such as vision, emotion, mood, language, and memory. Thus, acupuncture can activate brain regions related to cognitive function while simultaneously reducing blood pressure (Wang et al., 2016). This suggests that acupuncture therapy may provide a comprehensive effect by modulating the function of multiple brain regions, not only targeting blood pressure regulation but also positively impacting cognitive function. A study utilizing resting state functional magnetic resonance imaging (rsfMRI) investigated the protective effects of acupuncture on cognitive function in SHRs. Both electroacupuncture (EA) and manual acupuncture (MA) were found to protect cognitive function by modulating various brain regions, including the hypothalamus and the entorhinal cortex. Specifically, acupuncture influenced angiotensin secretion by astrocytes in the hypothalamus and brainstem, oxidative stress pathways, levels of pro-inflammatory cytokines, and arginine vasopressin secretion in the hypothalamus, as well as memory storage, consolidation, and reactivation processes in the entorhinal cortex.

In addition, acupuncture regulates neural functional connectivity between the hypothalamus, entorhinal cortex, and hippocampus, leading to a comprehensive regulatory effect on both blood pressure and cognitive function (Liu J. P. et al., 2023). Furthermore, acupuncture enhances the density and thickness of myelin in brain regions such as the corpus callosum, anterior cingulate cortex, and hippocampus via the JNK/GSK3β/NMDAR signaling pathway. It also increases the number and length of hippocampal neuronal spines, thereby mitigating white matter injury and cognitive decline associated with hypertension (Dong et al., 2024). These findings suggest that acupuncture positively influences cognitive function and brain structure through various pathways mediated by multiple brain regions, offering deeper insights into the multifaceted nature of acupuncture as a therapeutic approach.

### 4.2 Acupuncture improves cognitive function by modulating the microbial-gut-brain axis

Acupuncture can modulate the intestinal microbiota, promoting a shift toward a healthier composition, inhibiting inflammation, protecting the intestinal barrier, and playing a crucial role in the treatment of central nervous system disorders (Jiang et al., 2023). In a study on spatial learning and memory in aging rats treated with electroacupuncture, it was found that electroacupuncture could mitigate neuroinflammation by increasing the relative DNA abundance of Lactobacillus and Bifidobacterium, preventing lipopolysaccharide (LPS) translocation into the bloodstream, and inhibiting activated microglia as well as the TLR4/NF-κB signaling pathway (He C. et al., 2021). Acupuncture treatment in Parkinson’s disease (PD) mice significantly reduces the levels of Bacteroides, which previous studies have shown to secrete pro-inflammatory neurotoxins and stimulate macrophages and monocytes to release TNF-α via a polysaccharide-mediated pathway. It also restores the levels of Butyricimonas and influences the levels of metabolites, such as short-chain fatty acids (SCFAs) and/or lipopolysaccharide (LPS), which can contribute to neuronal dysfunction and cell death by affecting glial cells and pro-inflammatory factors (Jang et al., 2020). Electroacupuncture (EA) can increase the abundance of beneficial bacteria such as Lactobacillus, Dubotella, and Bifidobacterium in the Parkinson’s disease (PD) model. This, in turn, affects the gut microbiota through intermediaries such as circulating endotoxins, inflammatory mediators, oxidative stress, and lipid peroxidation, ultimately improving neurological function and alleviating motor symptoms in the model rats (Hu et al., 2024).

Studies have shown that acupuncture can have effects comparable to those of probiotics in regulating intestinal flora disorders in APP/PS1 mice and restoring the normal microbial structure. Specifically, acupuncture down-regulates Proteobacteria and Shigella, up-regulates Bacteroidetes, inhibits intestinal inflammation, and provides protective effects on the intestinal barrier. This protection is crucial for preventing the neuroinflammation commonly associated with Alzheimer’s disease (AD). The mechanism may involve reducing the abundance of Proteobacteria and Escherichia-Shigella, decreasing LPS load, and up-regulating the expression of tight junction proteins such as ZO-1 and occludin. In conditions of persistent intestinal flora disturbance, the beneficial effects of acupuncture on cognitive function and intestinal barrier function in APP/PS1 mice may be compromised (Hao et al., 2022). Additionally, acupuncture has been shown to lower LPS levels and reduce concentrations of inflammatory factors such as TNF-α and IL-1β. This reduction helps to decrease systemic inflammation caused by LPS stimulation and mitigates damage to the blood-brain barrier, ultimately leading to improved cognitive function (Zhang et al., 2022). These findings highlight the regulatory effects of acupuncture on intestinal flora, intestinal inflammation, and cognitive function, providing substantial support for the application of acupuncture therapy in neurological diseases.

### 4.3 The discussion of possibility and future research direction of acupuncture in treating hypertension with MCI by regulating the microbe-gut-brain axis

The microbial-gut-brain axis plays a crucial role in the pathogenesis and development of hypertension and MCI, with significant involvement of intestinal microbial metabolites, the nervous system, and the immune system. There is substantial evidence indicating that acupuncture can treat a range of conditions by modulating the intestinal microbiome, including irritable bowel syndrome (Yaklai et al., 2021), functional constipation (Yan et al., 2023), Parkinson’s disease (Jang et al., 2020), Alzheimer’s disease (Yan et al., 2024), atopic dermatitis (Yeom et al., 2024), obesity (Si et al., 2018), knee osteoarthritis (Wang et al., 2021), depression (Wang et al., 2024), and insomnia (Zhao et al., 2024). The regulatory mechanisms of acupuncture predominantly involve neural, immune, and microbial metabolite pathways. Additionally, some researchers have proposed exploring the potential of acupuncture for treating mild cognitive impairment through animal studies based on the brain-gut axis theory (Jin et al., 2022; Jin Y. et al., 2023). Given this evidence, this paper aims to investigate whether acupuncture can improve hypertension associated with MCI by regulating the microbe-gut-brain axis.

**FIGURE 3:**
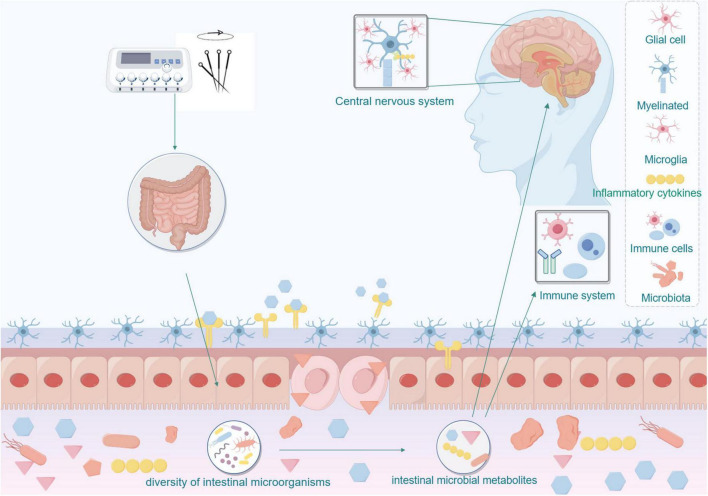
Acupuncture affects the nervous and immune systems by modulating the microbial-gut-brain axis.

Specifically, acupuncture may influence neurological pathways, inflammatory factors, and signaling mechanisms by modulating intestinal microbiota as shown in Figure 3. It may directly impact the nervous system or indirectly affect the immune system through microbial metabolites, thereby reducing blood pressure and enhancing brain function to improve cognitive performance. This research direction holds promise for deepening our understanding of the mechanisms underlying acupuncture treatment and offers a novel approach to exploring acupuncture’s application in managing Mild cognitive impairment in Hypertension.

## 5 Conclusion

In summary, exploring the microbial-gut-brain axis to understand the mechanisms underlying Mild cognitive impairment in Hypertension and related treatments has emerged as a prominent research focus. Existing studies have demonstrated that the microbial-gut-brain axis is intricately linked to both hypertension and MCI. Gut microbes influence blood pressure and cognition-related brain regions either directly or indirectly through metabolites, the RAS, sympathetic nerves, hippocampal pathways, immune cells, and inflammatory factors. Significant correlations have been observed between changes in gut microbiota abundance and both blood pressure and cognitive function. Therefore, investigating the mechanisms associated with intestinal microbiota can enable more precise and sensitive interventions to manage Mild cognitive impairment in Hypertension, potentially delaying or even preventing the progression from MCI to dementia.

Acupuncture, a widely recognized non-pharmacological therapy, has been shown to interact closely with the microbial-gut-brain axis. However, according to the current literature, there is still a lack of clinical trial and research data regarding how acupuncture improves hypertension and mild cognitive impairment (MCI) through the modulation of the gut microbiome.

Therefore, this mechanism still requires verification from multiple perspectives and dimensions. The evidence presented in this paper suggests that acupuncture can influence the abundance of gut microbiota and the levels of microbial metabolites associated with hypertension and mild cognitive impairment (MCI). Future studies can mainly demonstrate whether acupuncture and moxibustion can further regulate gene expression, protein level and various signaling pathways by improving the abundance of intestinal flora and the content of microbial metabolites, that is, indirectly regulate nerve, endocrine, immune and inflammatory factors by influencing intestinal microbes to treat hypertension complicated with MCI. Such studies will enhance our understanding of acupuncture’s mechanisms in treating Mild cognitive impairment in Hypertension and provide valuable evidence and direction for future clinical applications.
